# Vector surveillance of avian *Plasmodium* and West Nile virus in *Culex* mosquitoes from Doñana, a UNESCO World Heritage Site

**DOI:** 10.1186/s13071-025-06961-2

**Published:** 2025-08-02

**Authors:** Josué Martínez-de la Puente, Juliana Tamayo-Quintero, María José Ruiz-López, Jesús Veiga, Santiago Ruiz, Ana Vázquez, Laura Herrero, Ramón C. Soriguer, Jordi Figuerola

**Affiliations:** 1 Estación Biológica de Doñana (EBD, CSIC), Seville, Spain; 2 Universidad de Antioquia, Medellín, Colombia; 3 Centro Nacional de Microbiología, Instituto de Salud Carlos III, Madrid, Spain; 4 Servicio de Control de Plagas, Diputación de Huelva, Huelva, Spain; 5 Ciber de Epidemiología y Salud Pública (CIBERESP), Madrid, Spain; 6 Ciber de Enfermedades Infecciosas (CIBERINFEC), Madrid, Spain

**Keywords:** Avian malaria, *Culex pipiens*, *Culex perexiguus*, *Dirofilaria*

## Abstract

**Background:**

Mosquito-borne pathogens produce relevant diseases causing human fatalities worldwide. In addition, mosquitoes transmit a variety of pathogens to livestock and wildlife, negatively affecting local economies and causing ecological impacts.

**Methods:**

Mosquitoes collected in a highly protected wetland from southern Spain were molecularly screened for the presence of three major pathogens, including the zoonotic flavivirus West Nile virus (WNV), avian *Plasmodium*, and filarioid nematodes.

**Results:**

Overall, 95 mosquito pools including 1376 females corresponding to 4 *Culex* species were molecularly analyzed, including 40 mosquito pools containing 390 *Culex pipiens*, 42 mosquito pools containing 880 *Culex perexiguus*, 10 mosquito pools containing 102 *Culex modestus*, and 3 mosquito pools containing 4 *Culex laticinctus*. WNV was detected in 5 *Cx. perexiguus* pools. Avian *Plasmodium* was found in 28 mosquito pools tested, including 17 pools of *Cx. perexiguus* and 11 pools of *Cx. pipiens*. Three different *Plasmodium* spp. lineages were found in mosquitoes, corresponding to the morphospecies: *P. vaughani* (SYAT05; *n* = 22), *P. matutinum* (LINN1; *n* = 4), and the *Plasmodium* sp. (SGS2; *n* = 1). One positive sample was not identified at the lineage level. *Plasmodium* prevalence was significantly associated with mosquito species and sampling session and marginally related with mosquito pool size. None of the pools tested were positive for the presence of *Dirofilaria* spp.

**Conclusions:**

These results represent the most taxon extensive survey of pathogens in mosquitoes in Doñana. This study expands the knowledge of the diversity of pathogens naturally circulating in this protected wetland in southern Spain. Recommendations for the population are considered.

**Graphical Abstract:**

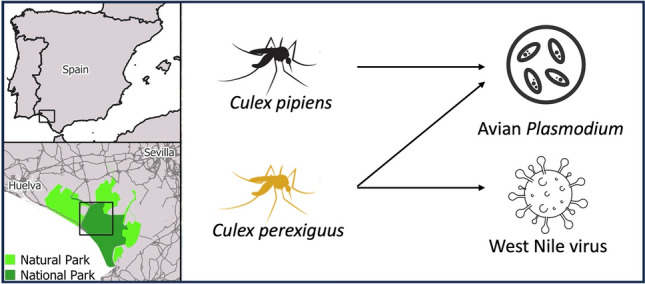

## Background

Mosquitoes are among the most relevant vectors of pathogens affecting wildlife, livestock, and human populations. Many of these pathogens are zoonotic, naturally circulating within wildlife via mosquito bites but potentially affecting humans. This is the case of West Nile virus (WNV), a mosquito-borne flavivirus that uses birds as its natural reservoirs. WNV-infected mosquitoes can transmit the virus to humans or horses, which act as accidental dead-end hosts [1]. Although sporadic human cases had been reported in Spain previously, the first large outbreak of West Nile disease occurred in 2020 in Andalusia and Extremadura, southern Spain, resulting in at least 77 human cases and 8 fatalities [2]. In 2024, 158 locally acquired human cases of WNV infection were reported in Spain, mostly in the southern part of the country, leading to 20 fatalities [3].

WNV cases in southern Spain are mainly associated with lineage 1, which has also been detected in mosquitoes [4, 5]. WNV antibodies have been found in wild birds and feral horses over the past two decades [6, 7]. During this period, bird exposure to WNV infections has occurred in natural and low-populated environments [8]. This includes the Doñana World Heritage Site, a protected wetland in southern Spain. Although a previous study conducted in 2010 did not detect WNV in mosquitoes from Doñana [9], seroprevalence surveys in feral horses sampled between 2005 and 2020 support the continuous WNV circulation in the area [6]. According to these results, WNV circulation may have increased in the area, as supported by the higher presence of WNV antibodies found in horses since 2020, when a prevalence of 25% was recorded [6]. These findings highlight the need for studies to identify the mosquito species involved in pathogen transmission in this protected area and to understand their role in potential changes in the epidemiology of WNV.

Moreover, mosquitoes transmit various parasites, including those that affect wildlife. Avian malaria parasites of the genus *Plasmodium* are widespread parasites naturally infecting birds on all continents except Antarctica. At least 55 morphospecies of avian *Plasmodium* are recognized [10], although the genetic diversity of these parasites is considerably higher [11]. Avian *Plasmodium* is transmitted by mosquitoes from an infected individual to a new host, with *Culex* mosquitoes playing a key role as natural vectors [12]. Studies assessing the diversity of avian *Plasmodium* in birds have been conducted extensively worldwide, revealing the impact of these parasites on wild birds [13] and birds held in captivity [14], and potentially contributing to the decline of common bird populations [15]. In southern Spain, *Plasmodium* infections have been confirmed in different bird species, mainly passerines [16, 17], but also water birds [18]. A considerable diversity of parasite lineages has been detected in house sparrows across 45 sampling sites in southern Spain, including Doñana, where 12 different lineages of avian *Plasmodium* were recorded, with a mean prevalence of 29.6% [16]. However, despite their importance in parasite transmission, information on the diversity of avian *Plasmodium* harbored by mosquitoes under natural conditions remains limited compared with data obtained from birds.

We developed a molecular xenomonitoring study, that is, the detection of pathogen genetic material in field-collected mosquitoes, to identify the diversity of pathogens potentially transmitted by mosquitoes in the Doñana Word Heritage Site. The mosquito species analyzed include *Culex pipiens* and *Culex perexiguus*, both of which are key vectors of bird pathogens, including WNV and avian malaria, in southern Spain [4, 19]. Given *Culex* species, including *Cx. pipiens*, may be also involved in the transmission of filarioid nematodes such as the zoonotic *Dirofilaria* in other regions of Spain [20, 21], we also tested mosquitoes molecularly for the presence of these parasites. *Dirofilaria* and other filarioids naturally circulate in the study area [22, 23], although the role of mosquitoes as vectors has been poorly investigated. This study could provide valuable insights into the potential exposure of humans and animals to mosquito-borne pathogens, including zoonotic ones, in Doñana and surrounding areas.

## Methods

### Mosquito collection and identification

**Fig 1.**
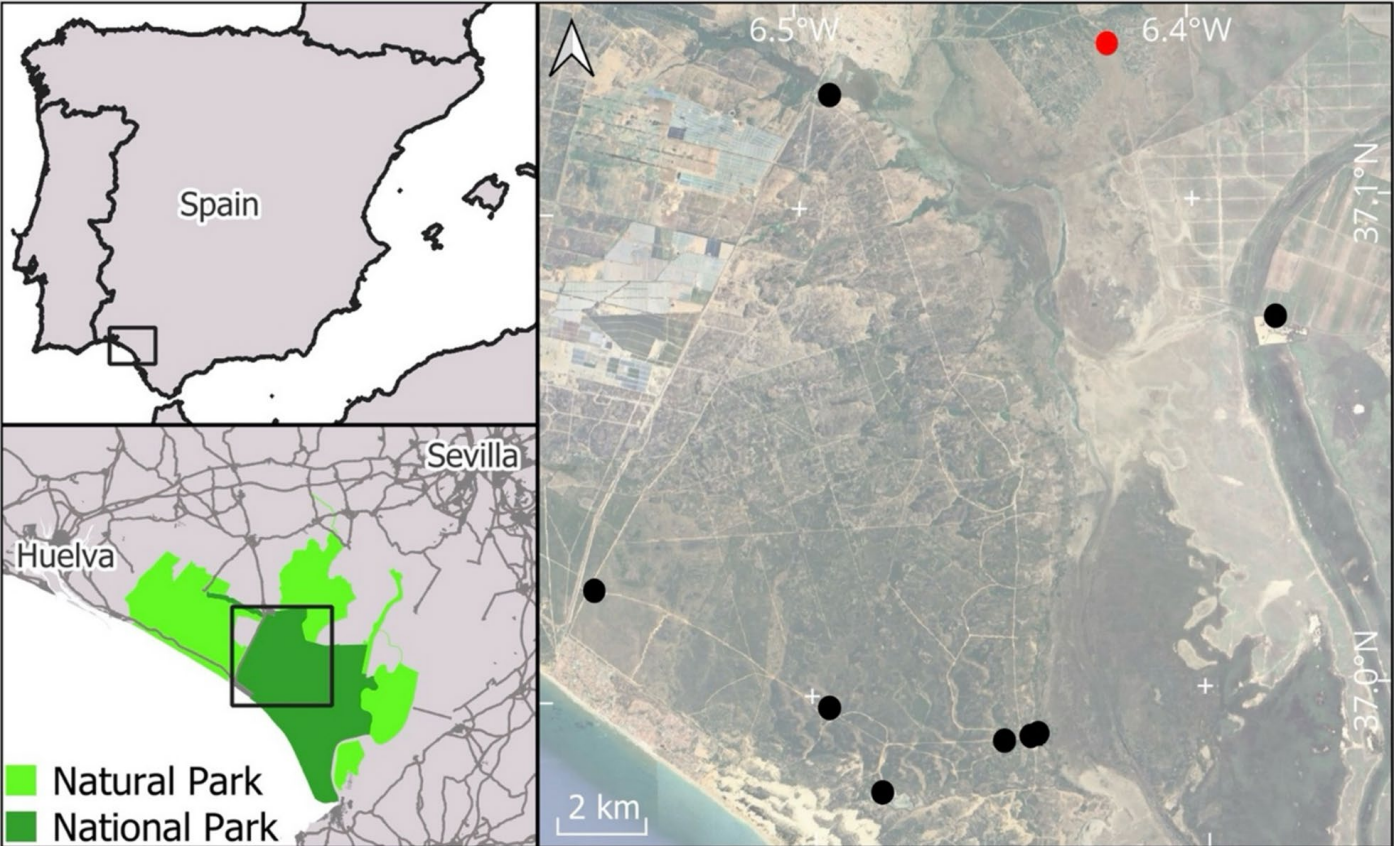
Sampling sites included in this study. Red circle indicates the area where West Nile virus was detected in mosquitoes. Note that two localities are in close proximity, causing the symbols to overlap

Mosquitoes were captured at nine sampling sites within Doñana National and Natural Park (Fig. 1). At each sampling site and during each sampling session, mosquitoes were trapped for 24 h using two BG-Sentinel traps supplemented with approximately 1 kg of dry ice as a source of CO_2_. Four trapping sessions were conducted during this study. Mosquitoes sampling was carried out approximately every 45 days, from April to September 2023. Further details of the procedures are shown in Martínez-de la Puente et al. [24].

Adult mosquitoes were preserved in dry ice in the field and stored frozen at −80 °C. Mosquitoes were sorted by sex and date of collection on a chilly table, and were identified to species level on the basis of their morphology [25]. Mosquito pools containing 1–50 females were grouped according to the species, sampling site, and session. Four species of the *Culex* genus were selected for this study, as they are considered major vectors of the pathogens tested.

### Molecular screening of parasites

RNA and DNA were simultaneously co-extracted from each shredded mosquito pool with the Maxwell® extraction robot and the Viral Total Nucleic Acid Purification kit (Promega, Madison, Wisconsin, USA), following manufacturer instructions. Detection of WNV was conducted using the RT-PCR procedure [4, 26]. The presence and identity of *Plasmodium* parasites were determined using the protocol developed by Hellgren et al. [27]. This procedure allows for the identification of *Haemoproteus* parasites using the same polymerase chain reaction (PCR) reactions, which are subsequently differentiated from *Plasmodium* by sequencing. *Leucocytozoon* parasites were not tested since they are transmitted by other insect vectors (i.e., Simuliidae). Positive amplifications were sequenced bidirectionally using the STAB-VIDA facilities (Lisbon, Portugal). Parasite lineages were identified using the Blast algorithm [28] in MalAvi and Genbank (NCBI) databases. The presence of filarioid nematodes was tested using the primer pair COIintF (5′-TGATTGGTGGTTTTGGTAA-3′) and COIintR (5′-ATAAGTACGAGTATCAATATC-3′) [29]. Negative and positive controls corresponding to avian *Plasmodium* and *Dirofilaria* were included in the lab procedures.

### Statistical analyses

Pathogen prevalence was calculated as a maximum likelihood estimate for variable pool size with the R package PoolTestR [30]. Further statistical analyses were restricted to the case of avian *Plasmodium*, as only this parasite reached detection rates high enough for the development of statistical tests. To identify factors associated with *Plasmodium* presence in mosquito pools, a generalized linear model (GLM) with a binomial distributed error and a logit link function was built including the infection status as the dependent variable (0: uninfected, 1: infected), sampling session (four levels) and mosquito species (*Cx. pipiens, Cx. perexiguus, Cx. modestus*) as factors, and pool size as a continuous independent variable. *Culex laticinctus* was excluded from the analysis due the low sample size (*n* = 3 mosquito pools). The overall effect of each variable in the fitted model were assessed using analysis of variance (ANOVA) type II. Statistical analyses were conducted in R 4.4.1 [31].

## Results

In total, we analyzed 95 mosquito pools including 1376 mosquito females corresponding to 40 mosquito pools containing 390 *Culex pipiens,* 42 mosquito pools containing 880 *Culex perexiguus*, 10 mosquito pools containing 102 *Culex modestus*, and 3 mosquito pools containing 4 *Culex laticinctus*. Five mosquito pools were positive for WNV, all of them corresponding to *Cx. perexiguus* (prevalence for *Cx. perexiguus* = 0.6%, 95% CI 0.2–1.4) collected at the Arroyo de la Cañada Mayor sampling site (Fig. 1; also known as *Cancela del Vicioso*) in the August and September trapping sessions.

Avian *Plasmodium* parasites were found in 28 mosquito pools (Table 1). Avian *Plasmodium* prevalence was significantly associated with mosquito species and sampling session, and marginally positively associated with pool size (mosquito species: likelihood-ratio-LR chi-squared = 9.85, *P* < 0.01; sampling session: likelihood-ratio (LR) chi-squared = 19.23, *P* < 0.01; pool size: LR chi-squared = 3.69, *P* = 0.05). Avian *Plasmodium* was found in 11 out of 40 *Cx. pipiens* pools (prevalence = 3.7%, 95% CI 1.9–6.3) and 17 out of 42 *Cx. perexiguus* pools (prevalence = 3.1%, 95% CI 1.8–4.9) while none of the 10 *Cx. modestus* pools tested were positive for the presence of parasites (prevalence = 0%, 95% CI 0–1.9). In addition, we found infected mosquitoes in all the trapping sessions except for August (April: prevalence = 4.8%; 95% CI 1.7–10.6; June: prevalence = 6.3%; 95% CI 3.0–11.3; August: prevalence = 0%, 95% CI 0–1.2; September: prevalence = 2.5%, 95% CI 1.4–4.2). Three different *Plasmodium* lineages were found in mosquitoes, with the *Plasmodium vaughani* lineage SYAT05 (*n* = 22) being the most commonly found, followed by the *Plasmodium matutinun* lineage LINN1 (*n* = 4) and the *Plasmodium* sp. lineage SGS2 (*n* = 1). One positive sample of *Cx. pipiens* was not identified to the lineage level due a poor-quality sequence. We did not find mosquito pools positive for *Haemoproteus* parasites.

**Table 1:** Number of mosquito pools analyzed in this study, according to mosquito species and sampling session

	April	June	August	September	Total	*Plasmodium* lineages
*Culex pipiens*	13 (5)	13 (5)	10 (0)	4 (1)	40 (11)	SYAT05 (*)
*Culex perexiguus*	5 (0)	7 (4)	11 (0)**	19 (13)**	42 (17)	SYAT05, LINN1, SGS2
*Culex modestus*	2 (0)	1 (0)	0 (0)	7 (0)	10 (0)	
*Culex laticinctus*	0 (0)	2 (0)	1 (0)	0 (0)	3 (0)	

The number of positive mosquito pools for avian *Plasmodium* are recorded in parentheses. (*) An additional unidentified parasite lineage was found in a single *Culex pipiens* pool. (**) Five *Cx. perexiguus* pools captured during the August and September trapping sessions were positive for WNV

Finally, none of the mosquito pools were positive for filaroid nematodes, although successful amplification of the positive control (e.g., DNA from *Dirofilaria*) was always recorded in the reactions.

## Discussion

We used a molecular xenomonitoring approach to identify the diversity of mosquito-borne pathogens, including viruses and parasites such as protozoa and nematodes, potentially transmitted by *Culex* mosquitoes in Doñana. This highly protected wetland hosts a rich diversity of wildlife, including birds and mammals, along with abundant mosquito populations that facilitate the local circulation of mosquito-borne pathogens.

This survey led to the detection of several pathogens circulating in the area, including zoonotic ones. WNV was detected in five *Cx. perexiguus* pools, further supporting the role of this species as a key vector of WNV in the region [32]. The first detection and isolation of WNV lineage 1 from *Cx. perexiguus* was reported in mosquitoes captured in the Guadalquivir marshes and adjacent wetlands during 2008–2009, with 7 positive mosquito pools out of 361 tested [5]. Additional records of WNV in *Cx. perexiguus* in southern Spain have been reported in recent years [4, 33, 34], although the virus was not detected in a previous extensive monitoring study conducted in Doñana [9]. The abundance of *Cx. perexiguus* correlates with the seroprevalence of WNV antibodies found in wild bird captured at the same sites [8]. Given the blood-feeding pattern of *Cx. perexiguus*, which includes both birds and mammals in its diet [32, 35], this species may act as a bridge vector of WNV from birds to horses. In Doñana, a recently published long-term survey of feral horses reported a mean prevalence of WNV antibodies of 8.1%, with interannual variations associated with climatic conditions [6].

Avian *Plasmodium* is a mosquito-borne parasite naturally infecting birds. Different mosquito species may be involved in its transmission, with species of the *Culex* genus playing a key role as vectors. In Spain, avian *Plasmodium* has been molecularly identified in different species of mosquitoes, including *Cx. pipiens* and *Cx. perexiguus* [36, 37], with studies also carried out in Doñana and surrounding areas [19, 38, 39]. Although the identification of *Plasmodium* DNA in mosquito pools does not confirm vector competence, the role of *Cx. pipiens* as a competent vector has been experimentally demonstrated [12, 40]. In contrast, the vector competence of *Cx. perexiguus* remains comparatively understudied. Our results support that avian *Plasmodium* prevalence varies among mosquito species, as they are similar in *Cx. pipiens* and *Cx. perexiguus* but lower in *Cx. modestus*. Further research is needed to elucidate the vector competence of these mosquito species in the region. Similarly, temporal differences in the prevalence of avian *Plasmodium* in mosquitoes may be due to factors related to the three main actors of this interaction: mosquitoes, birds, and parasites. In particular, high temperature during August may reduce the abundance of key mosquito vectors, potentially limiting parasite transmission. For example, the abundance of *Cx. pipiens* in wetlands of southern Spain, including Doñana, has been negatively associated with accumulated temperatures during the month prior to sampling [41]. Although *Cx. perexiguus* and *Cx. modestus* showed the opposite trend, their abundance in the area was considerably lower than that of *Cx. pipiens* [41].

We identified different *Plasmodium* lineages in mosquitoes from Doñana, including the *P. vaughani* lineage SYAT05, *P. matutinum* lineage LINN1, and lineage SGS2, which is not linked to any described morphospecies. Both SYAT05 and LINN1, as well as their corresponding morphospecies, are generalist parasites that infect birds [17] and mosquitoes [19, 37, 38] in southern Spain. Particularly, *Plasmodium* lineage SYAT05 has been reported in different bird species in Spain, including spotless starlings [42] and blackbirds [17], with the latter also being a common host of the LINN1 lineage in Spain and other European countries [43]. Lineage SGS2 was previously found in a single *Cx. pipiens* pool collected at a natural site in southern Spain [19].

Despite previous reports of *Dirofilaria* in *Cx. pipiens* from other regions of Spain [20, 21], we did not detect any filariod nematodes in the mosquitoes tested. Discrepancies among studies may reflect differences in host availability and parasite prevalence across areas with different landscape use. Domestic dogs are important reservoirs of *Dirofilaria* [44], which may occur in higher densities in rural and urban areas. Nevertheless, other wildlife species present in Doñana are known to be infected by *Dirofilaria* parasites, including the endangered Iberian lynx (*Lynx pardinus*) [22], which is a known host of *Anopheles atroparvus* [45], a potential vector of *D. immitis* in the Iberian Peninsula [46]. In addition, the recent record of *Aedes albopictus* in Doñana [24], which may also act as a vector of *D. immitis* [47], warrants further investigation into its potential role in the local transmission of parasites.

## Conclusions

We conducted an extensive molecular screening of pathogens naturally circulating in mosquitoes within the Doñana World Heritage Site, detecting the presence of the zoonotic WNV and avian malaria parasites. Our findings further support for the role of *Cx. perexiguus* as a major vector of WNV in natural ecosystems in southern Spain. In addition, this species and *Cx. pipiens* may play a key role in the local transmission of avian *Plasmodium*. Given the public health relevance of WNV, individuals visiting the area should reduce exposure to mosquito bites, consider use of repellents, and reduce their exposure to mosquito bites during the period of maximum activity of mosquitoes.

## Data Availability

Data supporting the main conclusions of this study are included in the manuscript.
